# Serum MicroRNA-21 as Marker for Necroinflammation in Hepatitis C Patients with and without Hepatocellular Carcinoma

**DOI:** 10.1371/journal.pone.0026971

**Published:** 2011-10-31

**Authors:** Verena Bihrer, Oliver Waidmann, Mireen Friedrich-Rust, Nicole Forestier, Simone Susser, Jörg Haupenthal, Martin Welker, Ying Shi, Jan Peveling-Oberhag, Andreas Polta, Michael von Wagner, Heinfried H. Radeke, Christoph Sarrazin, Jörg Trojan, Stefan Zeuzem, Bernd Kronenberger, Albrecht Piiper

**Affiliations:** 1 Department of Medicine I, University of Frankfurt/M., Frankfurt, Germany; 2 Institute of Pharmacology/ZAFES, University of Frankfurt/M., Frankfurt, Germany; Technische Universität München, Germany

## Abstract

**Background:**

MicroRNA-21 (miR-21) is up-regulated in tumor tissue of patients with malignant diseases, including hepatocellular carcinoma (HCC). Elevated concentrations of miR-21 have also been found in sera or plasma from patients with malignancies, rendering it an interesting candidate as serum/plasma marker for malignancies. Here we correlated serum miR-21 levels with clinical parameters in patients with different stages of chronic hepatitis C virus infection (CHC) and CHC-associated HCC.

**Methodology/Principal Findings:**

62 CHC patients, 29 patients with CHC and HCC and 19 healthy controls were prospectively enrolled. RNA was extracted from the sera and miR-21 as well as miR-16 levels were analyzed by quantitative real-time PCR; miR-21 levels (normalized by miR-16) were correlated with standard liver parameters, histological grading and staging of CHC. The data show that serum levels of miR-21 were elevated in patients with CHC compared to healthy controls (*P*<0.001); there was no difference between serum miR-21 in patients with CHC and CHC-associated HCC. Serum miR-21 levels correlated with histological activity index (HAI) in the liver (r = −0.494, *P* = 0.00002), alanine aminotransferase (ALT) (r = −0.309, *P* = 0.007), aspartate aminotransferase (r = −0.495, *P* = 0.000007), bilirubin (r = −0.362, *P* = 0.002), international normalized ratio (r = −0.338, *P* = 0.034) and γ-glutamyltransferase (r = −0.244, *P* = 0.034). Multivariate analysis revealed that ALT and miR-21 serum levels were independently associated with HAI. At a cut-off dC_T_ of 1.96, miR-21 discriminated between minimal and mild-severe necroinflammation (AUC = 0.758) with a sensitivity of 53.3% and a specificity of 95.2%.

**Conclusions/Significance:**

The serum miR-21 level is a marker for necroinflammatory activity, but does not differ between patients with HCV and HCV-induced HCC.

## Introduction

Hepatocellular carcinoma (HCC) is one of the most common solid tumors, rated third in mortality worldwide [1]. Most HCCs develop in fibrotic or already cirrhotic liver which are a result of chronic infection with hepatitis B (HBV) or hepatitis C virus (HCV) [1]. Currently, there are no biomarkers for the early detection of HCC, and most patients with HCC are diagnosed at advanced stages, which are associated with poor prognosis and low survival rates due to a lack of curative treatment options. α-Fetoprotein (AFP) has mainly been used for diagnosis of HCC; however, its sensitivity and specificity are not satisfying [2].

MicroRNAs (miRNAs) are small noncoding RNAs (18–24 nucleotides) that interact with their target mRNAs to inhibit translation by promoting mRNA degradation or to block translation by binding to complementary sequences in the 3′-untranslated region of mRNAs [3]. An integral role of miRNAs in cancer pathogenesis has begun to emerge. MiRNA expression profiling reveals characteristic signatures for many tumor types [4]. Importantly, miRNAs have also been detected in human serum and plasma, where they are remarkably stable [5], raising the possibility that unique miRNA patterns in serum and plasma might be used as non-invasive disease markers. In support of this, differences have been found between miRNA patterns in serum or plasma of patients with a number of malignancies and healthy controls [5].

Analysis of the miRNA signatures of a large number of tumor samples, including lung, breast, stomach, prostate, colon, and pancreatic cancer and their respective normal adjacent tissue, revealed that miR-21 is the only miRNA upregulated in all these tumors [4], including HCC [6]–[9]. Considerable evidence supports the hypothesis that miR-21 is a central oncomiR [10]. miR-21 levels are also significantly elevated in serum/plasma of patients with diffuse large B-cell lymphoma, ovarian cancer, prostate cancer or breast cancer [11]–[13], rendering it an interesting candidate as serum/plasma marker for malignancies.

miR-21 has also been linked to fibrosis in the lung [14], heart [15] and liver [9]. In the liver the level of miR-21 correlates with hepatic fibrosis [9]. A close link between miR-21 and hepatic fibrosis is supported by the findings that transforming growth factor β (TGF-β), a critical mediator of hepatic fibrogenesis [16], [17], promotes the expression of miR-21 [18], and that miR-21 decreases the expression of SMAD7 [9], a negative regulator of TGF-β signalling [17]. A recent study has reported that circulating miR-21 might be useful as biomarker for HCC [19].

Alterations of the miR-21 concentration in serum or plasma of patients with HCV-induced chronic hepatitis C (CHC) have not yet been reported. Hypothesizing that miR-21 serum levels might be related to either malignancy, i. e. HCC, or liver fibrosis/cirrhosis or both, we here investigated the relation between miR-21 serum levels and clinical parameters in patients with CHC, CHC plus HCC, and healthy volunteers.

## Methods

### Patients

Patients with CHC (n = 62) as well as CHC plus HCC (n = 29), who had undergone liver biopsy for staging and grading of CHC at the Frankfurt University hospital, were prospectively enrolled in the present cohort study. An independent cohort of 47 CHC patients was used for validation. Inclusion criteria were detectable anti-HCV antibodies and HCV-RNA for at least six months. Exclusion criteria were decompensated liver disease, malignancy other than HCC, organ transplantation, co-infection with HIV or HBV, immunosuppression and autoimmune co-morbidities. Patients' characteristics are summarized in Table 1 and 2. The Ethics Committee of the University Hospital Frankfurt approved this study. From all enrolled patients and healthy subjects written informed consent was obtained according to the Declaration of Helsinki.

**Table 1:** Characteristics of CHC patients, CHC patients with HCC and healthy subjects.

		CHC patients (n = 62)	CHC patients with HCC (n = 29)	Healthy subjects (n = 19)
**Age** (years)	Mean ± SD	46.1±11.2	61.4±9.1	33.2±10.6
**Sex** (n)	Male	34 (54.8%)	22 (75.9%)	11 (57.9%)
	Female	28 (45.2)	7 (24.1%)	8 (42.1%)
**HAI** (n)	2	7 (11.3%)	-	
	3	8 (12.9%)	-	
	4	13 (21.0%)	-	
	5	10 (16.1%)	2 (6.9%)	
	6	14 (22.6%)	1 (3.4%)	
	7	5 (8.1%)	2 (6.9%)	
	8	2 (3.2%)	2 (6.9%)	
	9	3 (4.8%)	2 (6.9%)	
	11	-	1 (3.4%)	
	Unknown	-	19 (65.5%)	
**Fibrosis** (n)	F0	7 (11.3%)	-	
	F1	20 (32.3%)	-	
	F2	4 (6.5%)	-	
	F3	6 (9.7%)	-	
	F4	10 (16.1%)	3 (10.3%)	
	F5	4 (6.5%)	1(3.4%)	
	F6	5 (8.1%)	9 (31.0%)	
	Unknown	6 (9.7%)	16 (55.2%)	
**ALT** (n)	Elevated*	44 (71.0%)	24 (82.8%)	
	Normal	18 (29.0%)	5 (17.2%)	

*>35 IU/l female; >50 IU/l male.

**Table 2:** Characteristics of the independent validation cohort of CHC patients.

		CHC patients(n = 47)
**Age** (years)	Mean±SD	45.4±10.4
**Sex** (n)	Male	22 (46.8%)
	Female	25 (53.2%)
**ALT** (n)	Elevated*	34 (72.3%)
	Normal	13 (27.7%)

*>35 IU/l female; >50 IU/l male.

### Liver Histology

Hematoxylin-eosin stained sections of formalin-fixed, paraffin-embedded liver biopsies were reviewed by experienced pathologists for evaluation of fibrosis stages (F0 = no fibrosis - F6 = cirrhosis) and the histologic activity index (HAI) in the liver according to the Ishak criteria [20].

### Blood Sampling

10 mL of peripheral blood was collected at the time of liver biopsy (serum tubes, Sarstedt, Nümbrecht, Germany). Cellular components were removed by two consecutive centrifugation steps (1500 *g* for 10 min at 4°C and 2000 *g* for 3 min at 4°C, respectively). Sera were stored at −80°C until use.

### Detection of miRNAs by Quantitative Real-time Reverse-Transcription (RT)-PCR

RNA was isolated from 500 µL serum using Tri®ReagentLS (Sigma-Aldrich, St. Louis, MO), chloroform and the *mir*Vana™ RNA isolation kit (Ambion-ABI, Austin, TX). Total RNA was eluted in 100 µL and stored at −20°C. 5 µL of RNA was reverse transcribed with the TaqMan® miRNA reverse transcription kit and the TaqMan® miRNA assay specific RT primers for miR-21 or miR-16 according to the instructions of the manufacturer (ABI). Real-time PCR was performed with 3 µL of each cDNA on a StepOne™Plus Real-Time PCR System (ABI) in duplicates. The cycle treshold (C_T_) defines the number of PCR cycles required for the fluorescent signal to cross the threshold.

### Statistical Analysis

Data were analyzed using the BiAS software for windows (version 9.07). Statistical significance for correlations was determined using Spearman's nonparametric rank test. Differences between two groups were evaluated using the Wilcoxon-Mann-Whitney-U test. *P* values<0.05 were considered to be significant. For the multivariate analysis a logistic regression with Wald's test was used to evaluate whether minimal (HAI_A+B+C_≤3) vs. mild to severe (HAI_A+B+C_>3) necroinflammation correlated independently with the parameters. The criterion for elimination in the stepwise model was P>0.1.

## Results

**Figure 1:**
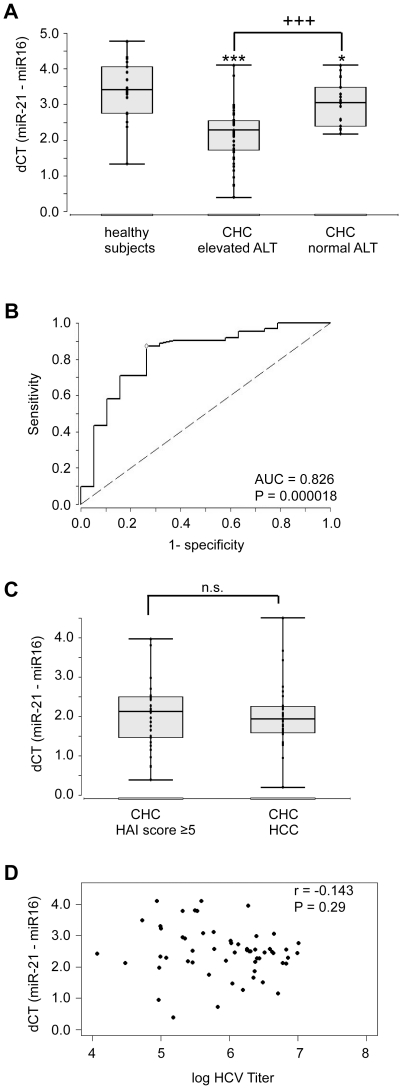
Increased serum miR-21 concentrations in patients with CHC and elevated levels of ALT. (A), dC_T_ values of miR-21 of sera from healthy control individuals (n = 19), patients with CHC and elevated serum ALT levels (n = 44) and CHC patients with normal serum ALT levels (n = 18). Boxes represent range, median and quartiles of the number of threshold cycles (C_T_) required to detect miR-21 by real-time RT-qPCR normalized to C_T_ of miR-16. Differences were calculated with Wilcoxon-Mann-Whitney-U-test. *P<.05, ***P<.001, compared with the healthy control group. ^+++^ *P*<.001 between CHC patients with normal or elevated ALT. (B), ROC curve analysis of serum miR-21 concentration for discriminating CHC patients and healthy controls. (C), dC_T_ values of CHC patients with elevated ALT and HAI≥5 (n = 31) and patients with CHC and HCC (n = 29) showing no significant difference in miR-21 levels (Wilcoxon-Mann-Whitney-U-test: *P*>.3) between both groups, n. s. = not significant. (D) Relationship between serum miR-21 levels and the serum HCV RNA in patients with CHC. The relation is not significant (*P* = .29).

miR-21 as well as miR-16, a miRNA found at constant levels in sera from various diseases, including chronic hepatitis B [5], [13], were quantified by real-time RT-PCR in sera from patients with CHC, CHC plus HCC and healthy controls. The mean C_T_ values (95% confidence interval (CI)) of miR-16 were 25.1 (24.5–25.6) in healthy donors and 25.2 (24.9–25.5), 25.0 (24.4–25.6) and 25.1 (24.2–26.0) in patients with CHC, in patients with CHC and normal alanine aminotransferase (ALT) and CHC plus HCC, respectively, showing that miR-16 in serum can be used as an internal control to normalize sampling variations in RT-qPCR in our collective of sera. The dC_T_ (C_T_-miR-21 - C_T_-miR-16) value negatively correlates with the serum level of miR-21. dC_T_ of miR-21 was higher in sera from healthy controls (3.4, CI: 3.0–3.8) than in sera from patients with CHC and elevated serum ALT activity (2.2, CI: 2.0–2.4) (*P*<0.001, Fig. 1A), a parameter reflecting liver damage. In contrast, the dCT value for miR-21 in sera from CHC patients with normal ALT values (3.0, CI: 2.7–3.3, *P*<0.05) only slightly differed from that of the control sera (Fig. 1A). ROC curve analysis was performed on the data from all CHC patients (without HCC) and controls. The area under the ROC curve (AUC) was 0.826 (CI: 0.711–0.942) (Fig. 1B), with a *P* value of 0.000018. The optimal cut-off value for miR-21 (normalized to miR-16) to discriminate between healthy controls and CHC patients was 3.16, with a sensitivity of 87.1%, specificity of 73.7% and false classification rate of 19.6%.

There was no significant difference between the dC_T_ values of miR-21 in sera from patients with CHC plus HCC and those with CHC and matched HAI (Fig. 1C). Moreover, we investigated if there is a relation between the serum levels of miR-21 and serum HCV RNA. However, there was no correlation between the serum miR-21 and HCV RNA level (*P* = 0.29) (Fig. 1D).

**Figure 2:**
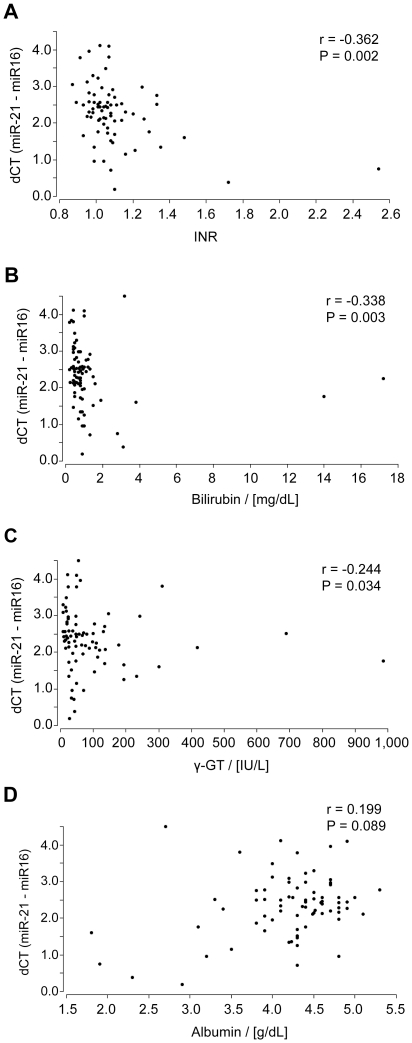
Relationship between serum miR-21 levels and INR (A), bilirubin (B), γ-GT (C) and serum albumin concentration (D).

To investigate the relation between the serum miR-21 level and standard liver function parameters, we correlated the relationship between the levels of miR-21 with the serum albumin concentration, international normalized ratio (INR), bilirubin and γ-glutamyl-transferase (γ-GT). There were positive correlations between the serum level of miR-21 and INR (r = −0.362, *P* = 0.002) (Fig. 2A), serum bilirubin concentration (r = −0.338, *P* = 0.003) (Fig. 2*B*) and γ-GT activity (r = −0.244, *P* = 0.034) (Fig. 2C). The correlation between the serum albumin concentration and the serum miR-21 level did not reach statistical significance (*P* = 0.0893) (Fig. 2D).

**Figure 3:**
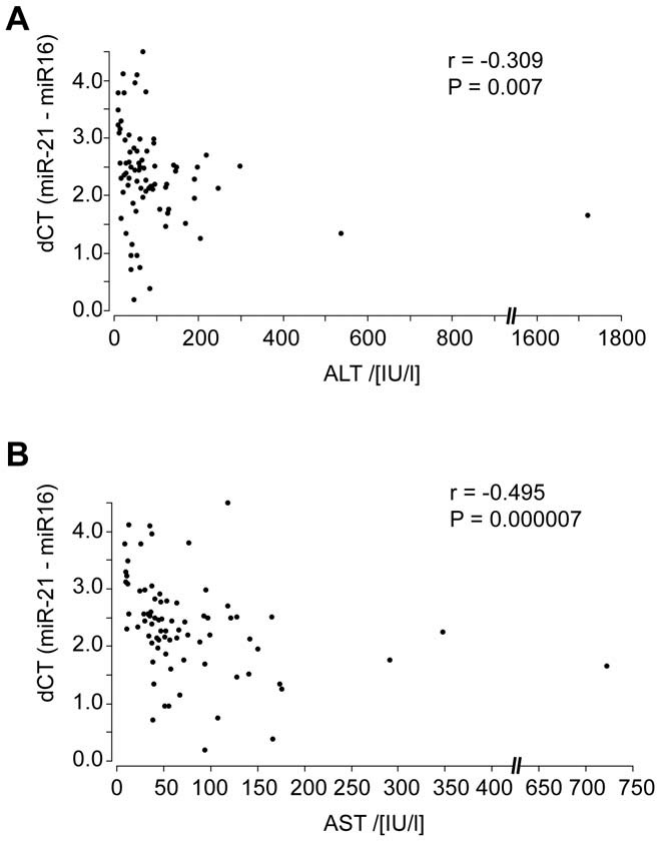
Correlation between serum miR-21 levels and ALT (A) or AST (B) in patients with CHC. Points represent dC_T_ values for miR-21 normalized to miR-16.

**Figure 4:**
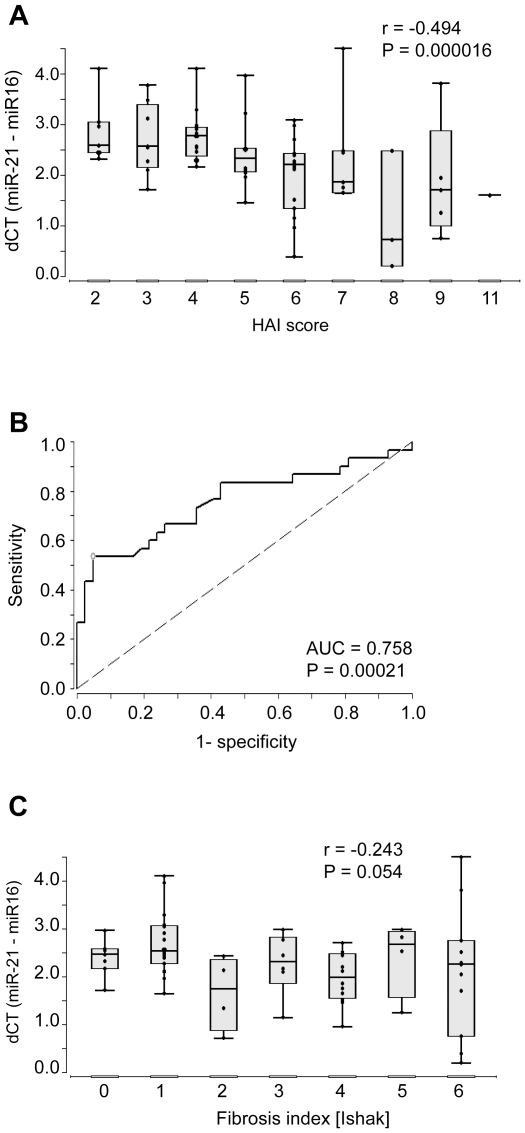
Relationship between serum miR-21 levels and the HAI score (A) ; (B), ROC curve analysis of serum miR-21 concentration for discriminating patients with minimal (HAI_A+B+C_≤3) vs. moderate to severe necroinflammatory activity (HAI_A+B+C_>3) using dC_T_ of miR-21; (C), relationship between serum miR-21 and fibrosis index in patients with CHC.

To examine if the levels of miR-21 in sera from patients with CHC as well as CHC plus HCC reflects necroinflammatory activity in the liver rather than HCC, we correlated the dC_T_ values of serum miR-21 with ALT, aspartate aminotransferase (AST) as well as with the histologic activity index (HAI) score in the liver. The dC_T_ value of miR-21 in the sera negatively correlated with serum levels of ALT (r = −0.309, *P* = 0.007) (Fig. 3A) and AST (r = −0.495, *P* = 0.000007) (Fig. 3B), i. e. the serum level of miR-21 positively correlated with ALT and AST activities. The dC_T_ values of serum miR-21 also strongly correlated with the HAI score (r = −0.494, *P* = 0.00002) (Fig. 4A).

**Figure 5:**
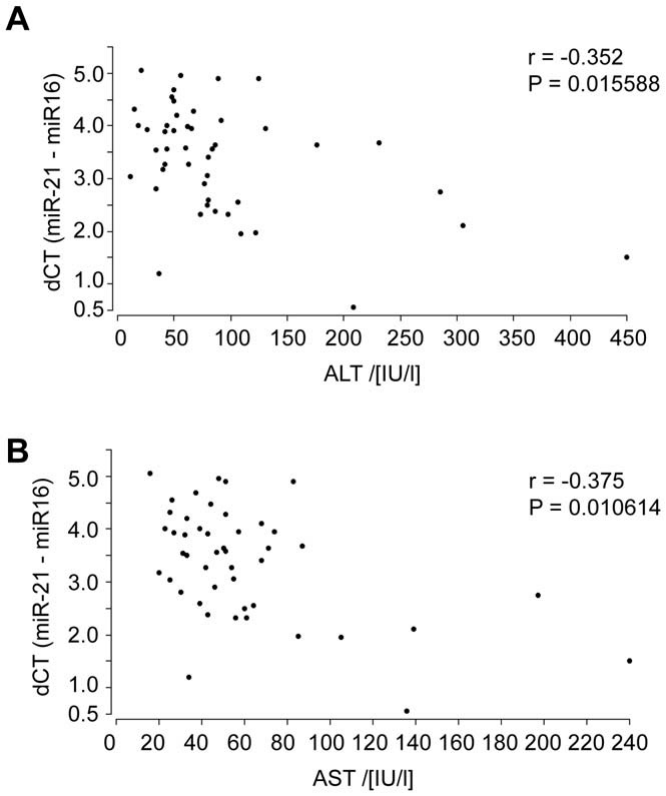
Validation of the relationship between the serum levels of miR-21 and ALT (A) and AST (B) in an independent cohort of 47 CHC patients.

In order to confirm the correlation between serum miR-21 levels and hepatic necroinflammation, we examined an independent validation cohort of 47 CHC patients. As illustrated in Fig. 5, the serum levels of miR-21 correlated with ALT (r = −0.352, P = 0.015588) and AST (r = −0.375, *P* = 0.010614). Histological classification according to Ishak was not available for these patients.

To investigate if the serum miR-21 level might be useful to discriminate patients with minimal (HAI_A+B+C_≤3) and mild to severe necroinflammation in the liver (HAI_A+B+C_>3), we performed multivariate analysis to examine the independency of the different parameters. Multivariate analysis revealed that only the correlations between HAI and serum ALT as well as miR-21 were independent (Table 3).

**Table 3:** Uni- and multivariate analysis of factors correlated to minimal (HAI≤3) vs. mild to severe (HAI>3) necroinflammation.

	Univariate analysis			Multivariate analysis		
	Odds ratio	95% confidence intervall	Wald's p-value	Odds ratio	95% confidence intervall	Wald's p-value
**Fibrosis stage**	1.4821	1.1150–1.9701	0.006744			
**Sex**	0.9524	0.3266–2.7774	0.928807			
**Age**	1.0817	1.0203–1.1469	0.008421			
**Log bilirubin**	1.7982	0.2684–12.0467	0.545413			
**Log ALT**	17.5591	2.4437–126.1671	0.004399	17.5478	1.3734–224.2070	0.027520
**Log AST**	36.3935.	3.3857–391.2003	0.003012			
**Log γGT**	2.4165	0.6476–9.0177	0.189115			
**INR**	19.1873	0.2329–1580.7251	0.18933			
**miR21-16**	0.1507	0.0399–0.5694	0.005262	0.1734	0.0487–0.6168	0.006804

The HAI score used for this analysis was the sum of HAI-A, -B and -C.

To test the suitability of miR-21 to discriminate minimal from mild to severe necroinflammation, we performed a ROC curve analysis on the data. The AUC was 0.758 (*P* = 0.00021) (Fig. 4B). At the optimal cut-off value of 1.96, the sensitivity was 53.3%, the specificity 95.2% and false classification rate was 25.7%.

To investigate if the serum miR-21 level might be related to liver fibrosis, we correlated the dC_T_ values of serum miR-21 and the histological fibrosis index in the liver. There was a trend towards a negative correlation between the Ishak fibrosis index and the dCT of miR-21 in the sera from CHC patients (r = −0.243, *P* = 0.054) (Fig. 4C).

## Discussion

Compared to the corresponding normal tissue miR-21 has been shown to be consistently elevated in malignant tumors, including HCC [4], [7]. Importantly, this miRNA is not only upregulated in association with oncogenesis, but can act as oncogenic miRNA [21]. It inhibits targets related to apoptosis and to transformation such as programmed cell death 4 and phosphatase and tensin homolog [6], [22]. miR-21 is also elevated in sera from patients with prostate carcinoma [12], mamma carcinoma [13], [22] and HBV-associated HCC [23], raising the possibility that it may serve as serum marker for malignant diseases. A recent study has suggested that circulating miR-21 is a marker for HCC [19]. However, that study did not allow differentiation between patients with liver cirrhosis and HCC. We report here that the level of miR-21 in sera from patients with CHC-associated HCC was elevated compared to healthy controls. However, we also detected increased levels of miR-21 in sera from patients with CHC without HCC. Indeed, sera from patients with matched HAI score and CHC showed similar miR-21 levels than sera from patients with CHC-associated HCC. Thus, the elevation of serum miR-21 levels in our CHC patients appears to be mainly associated with chronic hepatitis rather than HCC. The link between the elevated levels of serum miR-21 and CHC is supported by our finding that miR-21 serum levels strongly correlated with ALT and AST activities in two independent cohorts of CHC patients, parameters of ongoing liver damage. Concordantly, elevated serum levels of miR-21 were found only in CHC patients showing elevated ALT levels, whereas sera from CHC patients with normal ALT levels also contain normal miR-21 serum concentrations. Moreover, the serum miR-21 levels also strongly correlated with the HAI score, supporting the suggestion that the serum miR-21 level is related to necroinflammatory activity in the liver in patients with CHC. Multivariate analysis of the data from patients with minimal or mild to severe necroinflammation in the liver revealed that only ALT and miR-21 correlated independently with necroinflammation in the liver. Thus, the serum miR-21 concentration might be a useful parameter to differentiate patients with minimal vs. mild to severe necroinflammation in the liver which is of clinical relevance.

Mechanistically, miR-21 may leak from damaged cells similar to ALT and AST, or may be actively exported from altered tissues as a mechanism of adaptation to alterations of the state of a cell [24]. Recent evidence suggests that the majority of miRNAs in serum or plasma, including miR-21, is complexed to proteins such as Ago2, thereby explaining the high stability of endogenous miRNAs in serum/plasma [25].

There is clear evidence for a close relation between miR-21 and hepatic fibrosis. Thus, the hepatic level of miR-21 appears to correlate with the stage of liver fibrosis [9]. miR-21 is strongly expressed in tumor cells, but also in tumor-associated fibroblasts [26]. In the present study the miR-21 concentration in serum showed a strong correlation with the HAI score, whereas the relation between the serum miR-21 level and the fibrosis score did not reach statistical significance, which is in agreement with a study published during revision of the present manuscript [27]. This suggests that serum miR-21 levels are related to liver damage activity rather than to liver fibrosis in patients with CHC.

There was no correlation between the concentrations of miR-21 and HCV RNA in serum. This can be reconciled with the lack of correlation of HCV serum level and disease activity in patients with CHC [28].

Similar to the data of the present study, it has recently been reported that sera from HBV-induced chronic hepatitis contain elevated levels of miR-21 in comparison to healthy controls [23]. However, in another study with patients suffering from HBV-induced chronic hepatitis, no elevation of serum miR-21 levels was found despite strongly elevated ALT and HAI scores in these patients [29], leaving the role of serum miR-21 in HBV-induced chronic hepatitis unclear.

Recent studies reported differential levels of miR-16 in sera from patients with HCC, chronic hepatitis of different etiology and healthy controls [27], [30]. In the present study, however, we did not find differences in the levels of miR-16 between sera from patients with CHC, CHC-associated HCC and healthy controls. Our data are in agreement with several other studies reporting invariant levels of miR-16 in sera from patients with several diseases [13], including HBV-induced chronic hepatitis and HCC, and healthy controls [23], [29], prompting utilization of this miRNA for normalization [13]. The reasons for the different findings between the studies remain elusive.

Our recent data show that serum levels of the liver-specific miR-122 also correlate with the HAI score as well as serum AST and ALT levels [31]. Thus, miR-21 and miR-122 may originate from the inflamed liver. However, miR-21 and miR-122 differ in that miR-21, but not miR-122, correlates with bilirubin, INR and γ-GT in CHC patients. Obviously, the serum levels of miR-21 and miR-122 reflect overlapping, but not identical disease parameters in CHC patients. This might be related to the different expression pattern of miR-21 and miR-122, miR-122 being highly selective for the liver, whereas miR-21 shows significant expression in other cells and tissues. For instance, miR-21 is strongly expressed in lymphocytes [32]. Thus, release of miR-21 from several different cell types may contribute to the elevation of the serum miR-21 level in patients with chronic hepatitis. A drawback of the broader expression pattern of miR-21 compared to miR-122 is that co-morbidities are more likely to reduce the diagnostic power of the serum miR-21 level than that of miR-122.

The identification of non-invasive biomarkers for the diagnosis of diseases has become a rapidly growing area of clinical research [33]. The discovery of miRNAs circulating in the peripheral blood has opened new directions of research to identify new non-invasive ways of diagnosis of disease. In summary, the data of the present study indicate that the level of serum miR-21 is suitable to detect necroinflammatory activity in the liver and not the presence of HCC. It appears likely that serum miRNAs can provide information on different aspects of chronic hepatitis not available from the presently used routine parameters of liver damage.
